# Metabolomics changes in brain-gut axis after unpredictable chronic mild stress

**DOI:** 10.1007/s00213-021-05958-w

**Published:** 2022-02-08

**Authors:** Qiuyue Xu, Mingchen Jiang, Simeng Gu, Xunle Zhang, Guangkui Feng, Xianjun Ma, Shijun Xu, Erxi Wu, Jason H Huang, Fushun Wang

**Affiliations:** 1 School of Nursing, Nanjing University of Chinese Medicine, Nanjing, 210023 China; 2 Department of Pediatrics, Hospital of Nanjing University of Chinese Medicine, Nanjing, 210004 China; 3 Institute of Brain and Psychological Science, Sichuan Normal University, Chengdu, 610066 China; 4 Department of Psychology, Jiangsu University Medical School, Zhenjiang, 212013 China; 5 Department of Neurology, Lianyungang Hospital of Chinese Medicine, Nanjing University of Chinese Medicine, Lianyungang, 222000 China; 6 School of Pharmacy, Chengdu University of Traditional Chinese Medicine, Chengdu, 611137 Sichuan China; 7 Department of Neurosurgery, Baylor Scott & White Health, Temple, TX 76508 USA; 8 Department of Surgery, Texas A&M University College of Medicine, Temple, TX 76508 USA

**Keywords:** Major depressive disorders, UCMS, MDD, Brain-gut axis, Metabolomics

## Abstract

**Background:**

Major depressive disorder is a leading cause of disability worldwide, affecting up to 17 % of the general population. The neural mechanisms of depression, however, are yet to be uncovered. Recently, attention has been drawn to the effects of dysfunctional brain-gut axis on depression, and many substances have been suggested to be involved in the communication between the gut and brain, such as ghrelin.

**Methods:**

We herein systematically examined the changes of metabolomics after unpredictable chronic mild stress (UCMS)–induced depression-like behaviors in rats and compared the altered metabolites in the hippocampus and jejunum samples.

**Results:**

Our results show that many metabolites significantly changed with UCMS both in the hippocampus and jejunum, such as L-glutamine, L-tyrosine, hydroxylamine, and 3-phosphoglyceric acid. Further studies suggested that these changes are the reasons for anxiety-like behaviors and depression-like behaviors in UCMS rats and also are the reasons for hippocampal neural plasticity.

**Conclusions:**

Coexistence of brain and gut metabolic changes in UCMS-induced depressive behavior in rats suggests a possible role of brain-gut axis in depression. This study provides insights into the neurobiology of depression.

**Supplementary Information:**

The online version contains supplementary material available at 10.1007/s00213-021-05958-w.

## Introduction

Major depression is affecting more than 17% of populations worldwide (Otte et al. 2016; Putnam et al. 2017), and the World Health Organization anticipated that depression will become the first health problem in 2020 (Gu, et al. 2020). Despite recent research advances, fewer than half of major depressive disorder (MDD) patients respond to antidepressant medications, thus highlighting the need to characterize the specific pathophysiology of MDD. However, the majority of previous studies have focused on the brain-specific mechanism and circuits, with only limited focus on the potential contributions of peripheral organs (Qin et al. 2019). Recently, attention has been drawn to the brain-gut axis, and the association between gut-brain axis with depression has been recognized. The brain-gut axis is a bidirectional, multifaceted communication system (Cryan et al. 2019), and the gut and brain might affect each other via the enteric nervous system, the parasympathetic system, the immune system, and microbial metabolites (Duman et al. 2016). For example, the vagus nerve represents the main component of the parasympathetic nervous system and plays an important role in depression (Breit et al. 2018). Meanwhile, brain activities can also be regulated by brain-gut peptides, such as ghrelin and insulin that are released by the gut (Sharon et al. 2019).

More recently, the microbiota has emerged as a key player in control of the brain-gut axis, especially during conditions of stress provoked by real or perceived homeostatic challenge. The gut microbiota, microorganisms that reside in the gastrointestinal (GI) tract, can modulate the activities of the central nervous system. For example, it is found that transplantation of microbiota from depressed patients to germ-free rats led to depressive behaviors (Zheng et al. 2016). In addition, it is found that microbiota-based interventions can alter the monoamine neurotransmitters in the brain (Strandwitz 2018), including norepinephrine (NE), dopamine (DA), serotonin, gamma-aminobutyric acid, and glutamate and metabolites (Baj et al. 2019; Xu et al. 2020). Despite increasing evidence associating affective disorders with imbalanced brain-gut interactions, the molecular mechanism of the brain-gut axis has yet to be elucidated in neuropsychiatric disorders such as depression. We herein investigated the metabolic molecule changes in the brain and intestine using gas chromatography-mass spectrometry (GC-MS/MS) in unpredictable chronic mild stress (UCMS) rat model of depression. As one of the most advanced analytic methods, GC-MS/MS combines gas chromatography and mass spectrometry to perform effective qualitative and quantitative analysis within a biological specimen. In this study, we used GC-MS to investigate the metabolites, especially those associated with the neurotransmitter metabolism in the hippocampus and intestines, and found many metabolites have significant change in both the brain and the intestines after introduction of UCMS to rats. These metabolic alterations are closely associated with neuroplasticity changes in the hippocampus, which could be implicated in the pathogenesis of depression.

## Materials and methods

### Animals and reagents

Depression occurrence in female patients is twice as many as male patients, because they are susceptible to hormone changes during the menstrual circle. This might the reason that a majority of stress models are using male rodents instead of females (Gu et al. 2018a). In this study all male Sprague-Dawley (SD) rats (weighing between 200 and 220 g, age, 6–8 weeks) were purchased from Nanjing Qing Longshan experimental animal company (Nanjing, China). Rats were kept under standard conditions. The animals were placed under a 12/12-h light/dark cycle (7 am/7 pm) and the prescribed temperature conditions (22 ± 2 °C). Food and water were provided to be accessed freely. All animal experiments are conducted in accordance with Guide for the Care and Use of Laboratory Animals (the National Research Council of the National Academies, Eighth Edition, 2011 revision) and carried out in accordance with the NIH Guide for the Care and Use of Laboratory Animals (NIH Publication No. 85-23, 1985, revised 1996) and also performed strictly under the institutional guidelines of the Animal Care and Use Committee of Nanjing University of Chinese Medicine (Nanjing, China).

Methoxyamine hydrochloride, pyridine, N,O-Bis(trimethylsilyl)trifluoroacetamide (BSTFA) with 1% trimethylchlorosilane (TMCS), fluoxetine, and 1,2-^13^C_2_-myristic acid were purchased from Sigma-Aldrich (St. Louis, MO, USA). N-hexane was purchased from ROE Scientific (St. Louis, MO, USA). Methanol of MS grade was supplied by Merck Millipore (Billerica, MA, USA). Ultrahigh-purity water was prepared by Millipore-Q system (Millipore Corporation, Billerica, MA, USA).

### Unpredictable chronic mild stress (UCMS) procedure

Iqbal et al compared subchronic variable stress (SCVS) and unpredictable chronic mild stress (UCMS) models to evaluate the susceptibility versus resilient phenotypes in male and female rats. SCVS induced depression-like behaviors in female rats only. The UCMS paradigm was more likely to induce depression-like behaviors in male rats (Iqbal et al. 2020). Based on the above, we replicated the UCMS model and involved experiments. Thirty-six Sprague-Dawley rats (SD rats) were randomly divided into three groups: group I, control group (control+0.9 % saline); group II, UCMS model of depression group (UCMS+0.9 % saline); and group III UCMS with fluoxetine (UCMS + fluoxetine 2.4 mg·kg^−1^). Group III started from the third week and were given continuous intragastric drug administration for 3 weeks continuously, 3 times a day. Fluoxetine solution was dissolved in 0.9 % saline (Gu et al. 2018b). Rats in group II and group III were exposed to consecutive UCMS for 5 weeks, while rats in group I were exempt. In summary, rats were exposed to the following stressors for each of the 24-h period: wet bedding (24 h), tail pinch (5 min), swimming in cold water (4 °C, 5 min), restraint (4 h), food or water deprivation (24 h), light/dark cycle inversion (24 h), cage tilting (4 h), and swimming in hot water (42 °C, 5 min).

### Behavioral tests and serum corticosterone test

After the UCMS procedure, the rats were tested with sucrose preference test (SPT) which was performed on day 35, open field test (OFT) on day 36, and forced swim test (FST) on day 37, as we previously reported (Gu et al. 2018a). All behavior tests were performed between 9:00 am and 4:00 pm by an observer who was blinded to the treatment groups (Gu et al. 2018b). The FST experiments were carried out between 9:00 am and 12:00 pm, in a plastic bucket (50 cm high and 25 cm diameter) containing 30 cm of water at a temperature of around 25 °C. The rats of each group were individually placed in the bucket for 5 min. The experiments were recorded with the Depression Scan software, which automatically analyzed the immobile time of each group of rats. The rats were blow-dried and put back into the cage after each experiment. The open field test is a depression-like behavior test that lasted for 5 min. The rats were placed in the center square of an open filed box (60 × 60 × 40 cm) which was divided into nine squares. The parameters used for analysis included the total travel distance, time spent in the central area (immobility time), and the grooming times. The sucrose preference test is a reward-based test to examine anhedonia. The rats were fastened for 20 h and then provided with two bottles, with one containing 2.5 % sucrose solution and the other containing pure water. The recording lasted for 2 h. The percentage preference was calculated according to the following formula: % preference = sucrose intake/(sucrose intake + water intake) × 100 %. Blood samples were obtained from the tail in the morning every Saturday to test plasma sample with enzyme immunoassay (EIA) kits.

### Hippocampus pretreatment for metabolomics analysis

Sample preparation for GC-MS analysis was revised according to previous reports (Qian et al. 2019). Briefly, 20 mg of frozen hippocampus was homogenized in 500 μL of MeOH, and 20 μL of ice methanol containing 1,2-^13^C_2_-myristic acid was added to a final concentration of 10 μg/mL and vortexed for 5 min. The mixture was centrifuged at 27400 × *g* for 10 min, and 300 μL of the mixture supernatant was dried at 45 °C and 15 kPa to evaporate MeOH. After drying, the sample was added to 50 μL of 15-mg/mL methoxyamine pyridine and vortexed for 5 min. Then, it was vortexed for 3 min and shaken at 30 °C for 90 min with the ThermoMixer C (Eppendorf, Hamburg, Germany). Immediately, thereafter, 50 μL of BSTFA trifluoroacetamide (BSTFA) containing 1 % TMS was added, and the sample was shaken again at 37 °C for 0.5 h. The mixture was then transferred to a sampler vial and subjected to GC-MS analysis.

### Jejunum pretreatment for metabolomics analysis

We chose jejunum to represent gut samples for analysis according to previous reports. The jejunum begins with the duodenal jejunum curve, especially the upper third is the most abundant part of the folds, the most important part of nutrient absorption, and the most important part of blood supply. Changes in jejunum mRNA expression can trigger neuromodulation and endocrine-related pathways and play a key role in metabolic-related diseases (Liang et al. 2018). Similarly, researchers are attempting to harness the advantages of the gut-brain axis to prevent neurocognitive disorders by enhancing intestinal health; the conclusion was drawn by focusing on the relevant indicators of jejunum samples (Wang et al. 2020). A 50-mg jejunum was added to 1-mL MeOH and grounded for 15 min; afterward, 20 μL of methanol (containing 12.5 μg/mL 1,2-^13^C_2_-myristic acid) was added for vortexing for 3 min to precipitate the protein and mix the internal standard with the sample. The mixture was centrifuged at 27400 × *g* for 10 min at 4 °C, and 300 μL of 300 supernatants was dried at 45 °C and 15 kPa to evaporate MeOH. After drying, the sample was added to 50 μL of a concentration of 15-mg/mL methoxyamine pyridine and vortexed for 5 min, and then the sample was reconstituted by shaking at 30°C for 90 min with the ThermoMixer C (Eppendorf, Hamburg, Germany). Immediately after, 60 μL of BSTFA trifluoroacetamide containing 1% TMS was added, vortexed for 1 min, and shaken again at 37 °C for 0.5 h. Subsequently, 60 μL of the supernatant was centrifuged at 45300 × *g* for 10 min and then injected into the GC-MS for analysis. All derivatization must be carried out in an anhydrous environment. Quality control (QC) samples of the hippocampus and jejunum were also prepared and derivatized according to the abovementioned protocol and were run after every 12 samples to monitor the retention time and elution order of the metabolites.

### GC-MS conditions for metabolomics analysis

Metabolomics analysis of hippocampus and jejunum samples was performed using a Thermo Trace 1310-TSQ 8000 gas chromatograph system coupled to a mass spectrometer. Each 1 μL of the derivative sample was injected onto a TG-5MS capillary column (0.25 mm × 30 m × 0.25 μm, Thermo Fisher, San Jose, CA, USA), and the excitation mode was separated by a ratio of 20:1. The gradient heating used helium (99.999%) as a carrier gas, which was maintained at a constant flow rate of 1.2 mL/min. GC temperature changes are as follows: 0–1 min, 60 °C; 1–14 min, 60–320 °C; and 14–19 min, 320 °C. The electron energy is 70 eV, and the MS data were obtained in full scan mode with a mass range of 50–500 m/z and a time range of 3.5–19 min. The QC was randomized by sequence, and a QC sample was injected between every 8 actual sample to check the peak intensity of the spiked internal standard (1,2-^13^C_2_-myristic acid).

### Electrophysiological tests

The rats were anesthetized with isoflurane and were checked for pain reflex. Then the rats were fixed on the dissection table to expose the heart. The injection needle was inserted into the right ventricle, to infuse 30-mL icy artificial cerebrospinal fluid (aCSF). And after the perfusion, the brain was decapitated immediately and was placed in ice-cold aCSF for 2 min. aCSF composition was 125 mM NaCl, 3.25 mM KCl, 1.25 mM NaH_2_PO_4_, 25 mM NaHCO_3_, 1mM MgCl_2_·6H_2_O, 2 mM CaC_l2_·2H_2_O, 11 mM D-glucose, and 2 mM ascorbate(pH 7.3-7.4), as previously reported (Wang et al. 2012, 2017). A total of 380-μm hippocampal slices were cut with Vibration slicer (Leica VT1000S), and the tissue was incubated in aCSF which was bubbled with air mixture of 95% oxygen and 5% carbon dioxide (Shi et al. 2018). Long-term potentiation (LTP) was assessed by recording field excitatory postsynaptic potentials in the hippocampus.

The whole perfusion system in vitro was continuously perfused with normal aCSF with 95 % oxygen and 5 % carbon dioxide. The temperature of cerebrospinal fluid was maintained at 24 °C and the flow rate was about 2–3 mL/min. A borate glass microelectrode filled with aCSF was used to record the evoked excitatory postsynaptic potentials. The concentric bipolar electrode (Bowdoin, ME 04287, USA) was placed in the Schaffer collateral branch to deliver presynaptic stimulation. The glass microelectrode was placed in the stratum radiatum of the CA1 area. The fEPSP corresponding to 30%–40% of the maximum amplitude response is tested. The stimulation electrode was used to deliver the test stimulus every 30 s, with each stimulus lasted 100 μs. After recording the stable baseline for at least 10 min, the high frequency stimulus program (HFS protocol consisting of 3 bursts of 100 Hz, 1 s pulses at 20 s intervals) was applied, and LTP was induced. LTP was recorded with the same baseline stimulation intensity (half of maximum stimulation) and recorded for at least 60 min. The slope of the induced fEPSP was measured and normalized to an average baseline of one minute. The field potential recording was filtered at 2 kHz, sampled at 20 kHz, and continuously recorded using a MultiClamp 700B amplifier (Axon Instruments, USA), and electrophysiological data was analyzed with Clampfit 10.5 software (Molecular Devices). The group allocations of the rats were blinded to the experimenters.

### Statistical analysis

All data are shown as the mean ± SEM. Differences in behavioral results were evaluated using Student’s *t*-test. All statistical analyses were carried out using SPSS software (version 20.0, SPSS Inc., USA). A *p* value less than 0.05 (*p*<0.05) was considered statistically significant. GC-MS data (Raw Data Files) of all samples were converted using the ABF converter (Ursell et al. 2014) (http://www.reifycs.com/AbfConverter) into MS-DIAL (v.2.7.2) software program (Furness et al. 2014) to perform peak detection, identification, and alignment. Metabolite identification was performed by comparing the MS spectrum and the Ankle RI index with those of the National Institute of Standards and Technology (NIST), and the metabolites were confirmed only when the EI similarity was higher than 80%. Normalized screening used R language after export (Kaelberer et al. 2018). The data were normalized to log conversion using MetaboAnalyst (http://www.metaboanalyst.ca/) before a series of multivariate statistical analyses, and the statistical significance of the identified metabolites was assessed using one-way ANOVA and *q*-test. The direct threshold changes of the final metabolite > 1.2 and *p*< 0.05 can be considered as potentially altered metabolites. All statistical analyses were then plotted using Prism 8 (GraphPad 8.00) and MetaboAnalyst 4.0.

## Results

### UCMS-induced remarkable depression-like behavior

**Fig. 1:**
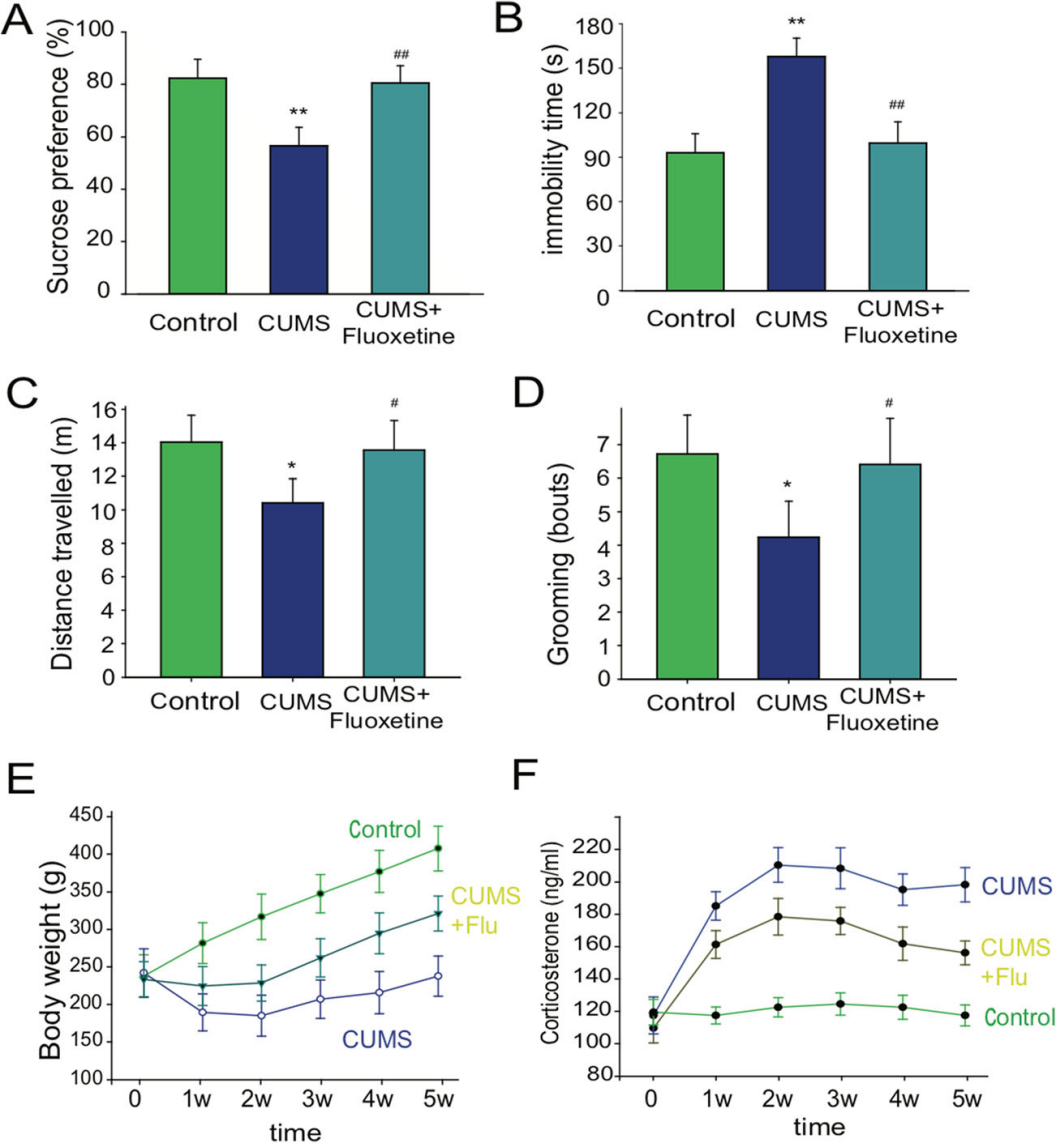
Behavior tests and physiological changes after UCMS. **A** Scores for sucrose preference test; **B** forced swim test; **C** open field test (distance); **D** open field test (grooming bouts). Sucrose preference test represents sugar water preference rates, which were calculated with the equation: SPT, sucrose consumption/(sucrose consumption + water consumption); **E** body weight changes during the UCMS training; **F** corticosterone (cort) levels in the blood (*n* = 12)

Adult male Sprague-Dawley (SD) rats were used to investigate the effects of chronic stress on the metabolic changes in both brain and gut. To determine if UCMS could successfully induce depression-like behaviors, we measured behavior changes, body weight, and plasma corticosterone (CORT) concentration of the rats after the UCMS training. Sucrose preference test, forced swim test (FST), and open field test (OFT) were used to test UCMS-induced depression-like behaviors. The results showed that UCMS significantly decreased the sucrose consumption in the sucrose preference test (SPT) (*p* < 0.01, compared with UCMS group; *p* < 0.01, compared with control group, one-way ANOVA, *n*=12, Fig. 1A). In the forced swim test, the immobility time in the UCMS group was significantly longer than that of the control group at the 5th week (*p <* 0.01 vs. control, one-way ANOVA, *n*=12, Fig. 1B). At the same time, the results of open field test (OFT) were also analyzed (Fig. 1C, D). Rats exposed to UCMS showed less total distance and fewer grooming bouts (*p <* 0.05), indicating that depression was successfully induced. In addition, we also monitored body weight and plasma corticosterone (CORT) over the UCMS procedure. Our results showed that UCMS significantly decreased body weight and increased the CORT, which is consistent with our previous reports (Fig. 1E, F) (Gu et al. 2018a, b). These results indicate that UCMS caused depression-like behaviors.

### Hippocampus and jejunum metabolomics profile and multivariate data

**Fig. 2:**
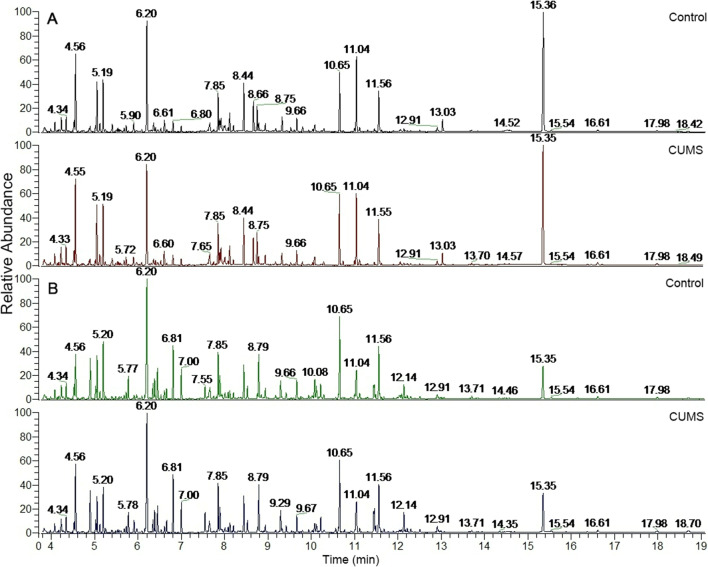
GC-MS total ion chromatograms (TIC) of hippocampus samples and jejunum samples. **A** The detected metabolites in the hippocampal samples can be called the typical total ion chromatogram (TIC) of the hippocampus (*n* = 8). In hippocampus samples, 144 endogenous metabolites were identified as changed significantly, including amino acids, glucose, amides, pyrimidines, and fatty acids. **B** In jejunum samples, 190 endogenous metabolites were identified as changed significantly, including amino acids, amino alcohol derivatives, fatty acid, and pyrimidines

We next investigated metabolic changes in UCMS-induced depressive behavior in rats. We tested low-molecular-weight metabolites with GC-MS/MS and expressed as chromatographic peaks, because these small molecules can be easily transported from the gut to brain. The detected metabolites in the hippocampal samples can be called the typical total ion chromatogram (TIC) of the hippocampus (Fig. 2A). In hippocampus samples, 144 endogenous metabolites were identified, including amino acids, glucose, amides, pyrimidines, and fatty acids. In jejunum samples, 190 endogenous metabolites were identified, including amino acids, amino alcohol derivatives, fatty acid, and pyrimidines. In addition, there were 4 metabolites which increased significantly in both hippocampus and jejunum.

### Metabolomics profile and multivariate data analysis

**Fig. 3:**
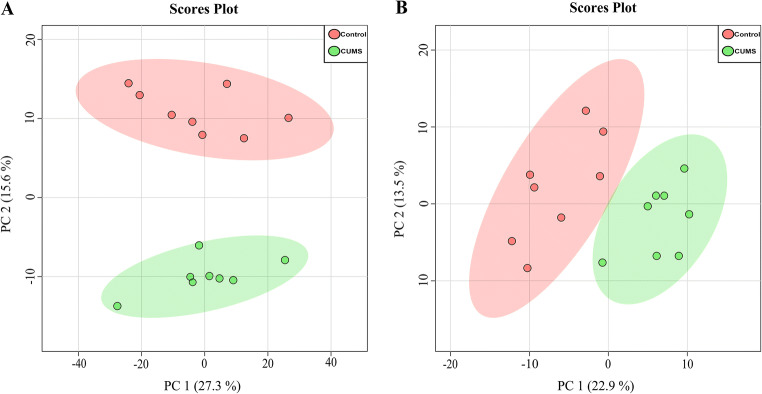
PCA scores scatter plot of hippocampus and jejunum metabolites. **A** Principal component analysis (PCA) score scatter plot demonstrated a good separation among UCMS group and control, indicating that UCMS significantly decreased some endogenous metabolites levels in hippocampus (*n* = 8). **B** PCA scatter plot of the metabolites changed in the jejunum of the UCMS rats (*n* = 8)

System stability and reproducibility analysis in both hippocampus and jejunum samples were performed based on GC-MS to obtain biochemical information on metabolites. The multivariate analysis method of principal component analysis (PCA) was done to investigate the similarities and differences among the groups. The PCA score scatter plot (Fig. 3) demonstrated significant difference among UCMS group and control group, indicating that UCMS significantly decreased some endogenous metabolite levels in the hippocampus (*p*< 0.05, *t*-test, *n*=8, Fig. 3A), and these metabolite similarly changed in the jejunum of the UCMS rats (*p*< 0.05, *t*-test, *n*=8, Fig. 3B). To evaluate system stability and reproducibility, PCA analysis was used to process data matrix of quality control (QC) samples. The PCA score plots of the hippocampus and jejunum samples showed that QC samples were clustered, verifying the stability in the GC-MS system throughout the analysis (Supplemental Fig. S1). In addition, the relative standard deviations (RSDs) of the peak height of 1,2-^13^C_2_-myristic acid was 13.64 % for the hippocampus and 7.58 % for the jejunum, indicating that the analytical conditions and sample process exhibited good repeatability and stability for the metabolomic study.

**Fig. 4:**
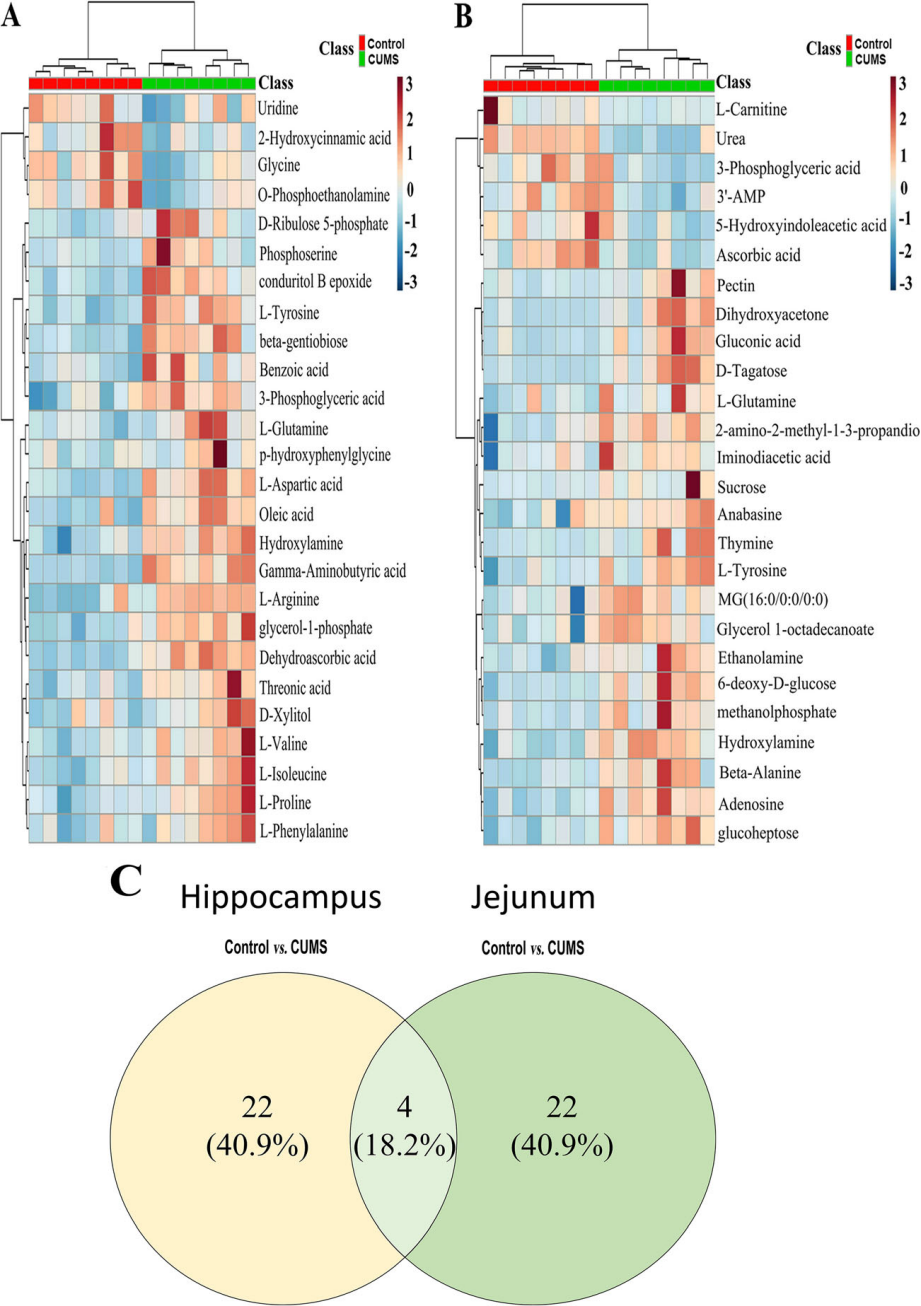
Heatmap of identified differential metabolites with hippocampus (**A**) and jejunum (**B**) metabolomics profile. Each cell in the heatmap represents the fold change of a particular metabolite. **C** A Venn diagram generated based on the proportion of the changed substances (*n* = 8)

There were 48 potential biomarkers in the hippocampus and in the jejunum that significantly changed by UCMS (Table 1), based on one-way ANOVA and *q*-test (*p* < 0.05), and there was a fold change >1.2 between the control and UCMS groups. The details of the altered metabolites in the hippocampus and jejunum samples are presented in Table 1. To illustrate the altered metabolites and the content difference among the three groups, metabolic heatmaps of hippocampus and jejunum were also plotted (Fig. 4A, B). It is worth noting there are more than 4 metabolites that changed more than 1.4-folds by UCMS both in the hippocampus and jejunum (Table 1).

**Table 1:** Potential biomarkers of the hippocampus and jejunum

No.	Retention time (min)	Metabolites	Human Metabolome Database	Kyoto Encyclopedia of Genes and Genomes	Sites	Fold change UCMS vs. control
1	4.3	L-arginine	HMDB0000517	C00062	HIP	0.65085^**^
2	5.77	L-valine	HMDB0000883	C00183	HIP	0.8308^**^
3	6	Benzoic acid	HMDB0001870	C00180	HIP	0.81405^**^
4	6.34	L-isoleucine	HMDB0000172	C00407	HIP	0.77481^**^
5	6.36	Gamma-aminobutyric acid	HMDB0000112	C00334	HIP	0.51652^**^
6	6.38	L-proline	HMDB0000162	C00148	HIP	0.79225^**^
7	6.45	Glycine	HMDB0000123	C00037	HIP	1.4292^**^
8	7.21	L-aspartic acid	HMDB0000191	C00049	HIP	0.57805^**^
9	8.05	Threonic acid	HMDB0000943	C01620	HIP	0.77119^**^
10	8.52	L-phenylalanine	HMDB0000159	C00079	HIP	0.81452^*^
11	9.09	D-xylitol	HMDB0002917	C00379	HIP	0.78283^*^
12	9.29	Glycerol-1-phosphate	NA	NA	HIP	0.68143^**^
13	9.32	p-hydroxyphenylglycine	NA	NA	HIP	0.72979^*^
14	9.42	O-phosphoethanolamine	HMDB0000224	C00346	HIP	1.4816^**^
15	9.71	Phosphoserine	HMDB0000272	C01005	HIP	0.65319^*^
16	9.8	Dehydroascorbic acid	HMDB0001264	C00425	HIP	0.69461^**^
17	11.06	Conduritol B epoxide	NA	NA	HIP	0.62354^**^
18	11.09	2-Hydroxycinnamic acid	HMDB0002641	C01772	HIP	1.2151^*^
19	11.18	D-Ribulose 5-phosphate	HMDB0000618	C00199	HIP	0.63592^**^
20	11.45	Oleic acid	HMDB0000207	C00712	HIP	0.81012^*^
21	12.56	Uridine	HMDB0000296	C00299	HIP	1.2632^*^
22	13.59	Beta-gentiobiose	NA	NA	HIP	0.31345^**^
23	5.03	Hydroxylamine	HMDB0003338	C00192	HIP and jejunum	0.78643^**^ and 0.73523^*^
24	9.32	L-glutamine	HMDB0000641	C00064	HIP and jejunum	0.72211^*^ and 0.76973^*^
25	9.54	3-Phosphoglyceric acid	HMDB0000807	C00597	HIP and Jejunum	0.73102^**^ and 2.2846^**^
26	9.95	L-tyrosine	HMDB0000158	C00082	HIP and jejunum	0.62828^**^ and 0.7132^*^
27	5.49	Methanolphosphate	NA	NA	Jejunum	0.38284^**^
28	5.86	2-amino-2-methyl-1-3-propanediol	NA	NA	Jejunum	0.76746^**^
29	5.91	Urea	HMDB0000294	C00086	Jejunum	4.0981^**^
30	6.03	Dihydroxyacetone	HMDB0001882	C00184	Jejunum	0.28812^**^
31	6.16	Ethanolamine	HMDB0000149	C00189	Jejunum	0.61592^**^
32	6.46	L-carnitine	HMDB0000062	C00318	Jejunum	2.6126^**^
33	6.61	Iminodiacetic acid	HMDB0011753	C19911	Jejunum	0.76514^*^
34	7.09	Thymine	HMDB0000262	C00178	Jejunum	0.5547^**^
35	7.26	Beta-alanine	HMDB0000056	C00099	Jejunum	0.59132^**^
36	8.53	Anabasine	HMDB0004350	C06180	Jejunum	0.56955^*^
37	8.85	Pectin	HMDB0003402	C08348	Jejunum	0.33909^**^
38	9.15	6-Deoxy-D-glucose	NA	NA	Jejunum	0.7039^**^
39	9.99	D-tagatose	HMDB0003418	C00795	Jejunum	0.25136^**^
40	10.31	Ascorbic acid	HMDB0000044	C00072	Jejunum	3.5443^**^
41	10.62	Gluconic acid	HMDB0000625	C00257	Jejunum	0.24617^**^
42	11.53	5-Hydroxyindoleacetic acid	HMDB0000763	C05635	Jejunum	1.41^*^
43	12.39	Glucoheptose	NA	NA	Jejunum	0.43798^**^
44	13	MG (16:0/0:0/0:0)	HMDB0011564	NA	Jejunum	0.70871^**^
45	13.27	Adenosine	HMDB0000050	C00212	Jejunum	0.60706^**^
46	13.38	Sucrose	HMDB0000258	C00089	Jejunum	0.3178^**^
47	13.71	Glycerol 1-octadecanoate	HMDB0031075	NA	Jejunum	0.72799^*^
48	14.55	3′-AMP	HMDB0003540	C01367	Jejunum	1.5942^*^

Note: ^*^p<0.05, ^**^p<0.01. *KEGG genome* Kyoto Encyclopedia of Genes and Genomes

### Trends in common differential metabolites in the hippocampus and jejunum

**Fig. 5:**
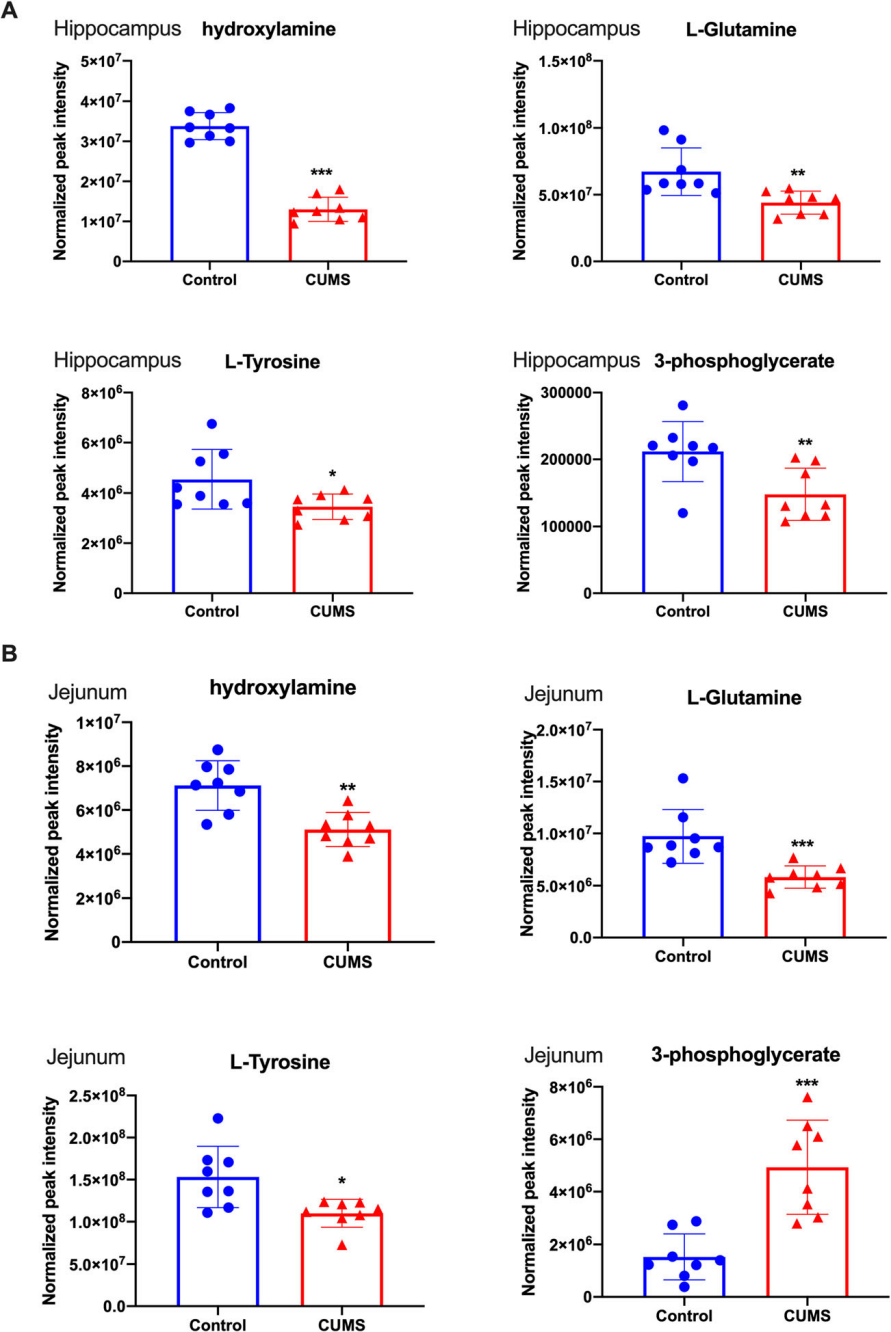
Scatter plots of significantly changed metabolites normalized peak intensity in rat hippocampus (**A**) and jejunum samples (**B**). The x-axis shows the specific metabolite’s normalized peak intensity, and each scatter represents a corresponding sample of the rat. represents hippocampus samples of control, represents hippocampus samples of UCMS, represents jejunum samples of control, represents jejunum samples of UCMS, the scatter plots show the mean and SD of the metabolites. *p<0.05, **p<0.01, vs. control (*n* = 8)

The quantification of common metabolites in the hippocampus and jejunum samples in UCMS-induced depression-like behavior in rats were analyzed (Fig. 5). Compared with those in the control group, the concentrations of many substances changed significantly (Table 1), for example, β-gentiobiose decreased to 0.31345 times in the UCMS group compared to normal control. Some substances increased, for example, L-carnitine increased to 2.6 times. However, the changes are different in the hippocampus and jejunum, the reason might be that the metabolites might be absorbed and transported in different format to the brain. There are four substances – (L-glutamine, L-tyrosine, 3-phosphoglyceric acid, hydroxylamine) – that showed a significant change in both the hippocampus and jejunum. This indicates that metabolism of the hippocampus and jejunum were similarly disturbed in UCMS-induced depression-like behavior in rats.

In order to verify the correlation between the jejunum and hippocampus, the metabolite profile of serum samples was also studied with GC-MS. A total of 101 endogenous metabolites were identified in serum samples. After the data was normalized, QC was used to test the stability of the samples (Fig. S2a), and the correlation between the groups was analyzed with principal component analysis. The normal group and the model group were significantly different (Fig. S2b). The metabolite changes were selected based on a change > 1.2 between the normal group and UCMS model group (Fig. S3). Consistently, there were 4 metabolites that can be identified; glutamine and L-tyrosine have metabolic changes in peripheral serum that are slightly different from those in the hippocampus and jejunum (Fig. S4).

### Neural plasticity changes in the hippocampus

**Fig. 6:**
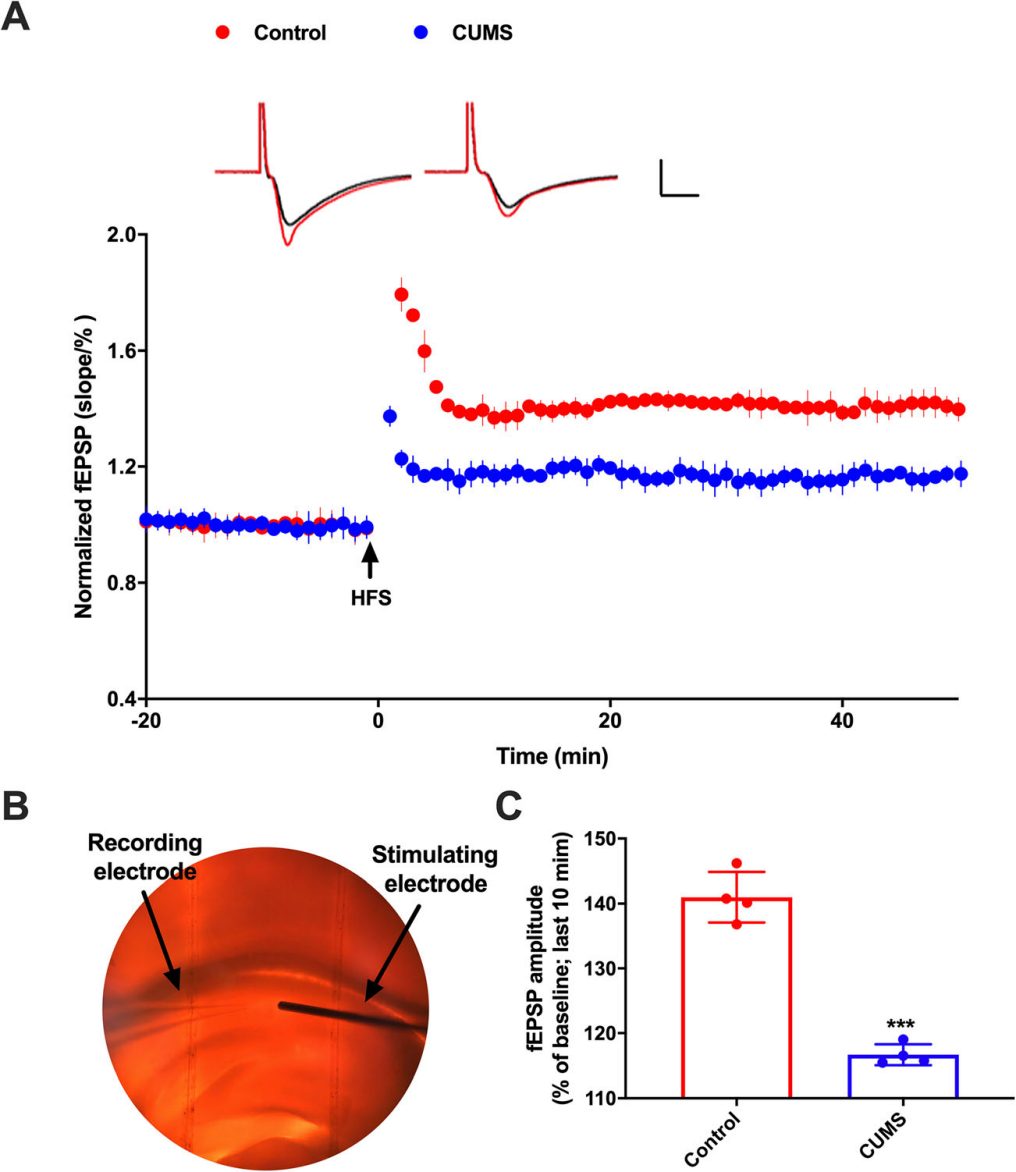
LTP results from the hippocampus. **A** LTP decreases in the hippocampus from UCMS mice (*n* = 4). **B** The record of the experiment operation. **C** The statistical analysis of the fEPSPs (field excitatory postsynaptic potentials). Unpaired *t*-test revealed that LTP was significantly decreased in hippocampal slices of UCMS rats. Data are presented as the mean ± SEM. *** *p* < 0.01, (*n* = 4)

We recorded LTP in CA1 region of the hippocampal slices of UCMS group rats and control group rats. LTP was successfully inducted and recorded (Fig. 6A) in normal artificial cerebrospinal fluid (aCSF). LTP was triggered by three high-frequency stimuli in both control group and in the UCMS group but was significantly different (calculation was done in 4 rats, and averaged amplitude of 10 EPSPs at 10–15 min was used for one animal) (Fig. 6B–C, * *p* < 0.01, *n*=4).

## Discussion

In this study, we performed a comprehensive analysis of metabolic changes in brain-gut axis of rats with UCMS-induced depression-like behavior. Our data show that UCMS-induced depression-like behavior was accompanied with many metabolic changes that similarly happened in both the hippocampus and intestine, such as hydroxylamine, L-glutamine, L-tyrosine, and 3-phosphoglyceric acid. These similar metabolite changes in the jejunum and hippocampus confirm that the brain-gut axis is closely related to the pathogenesis of depression-like behavior.

**Fig. 7:**
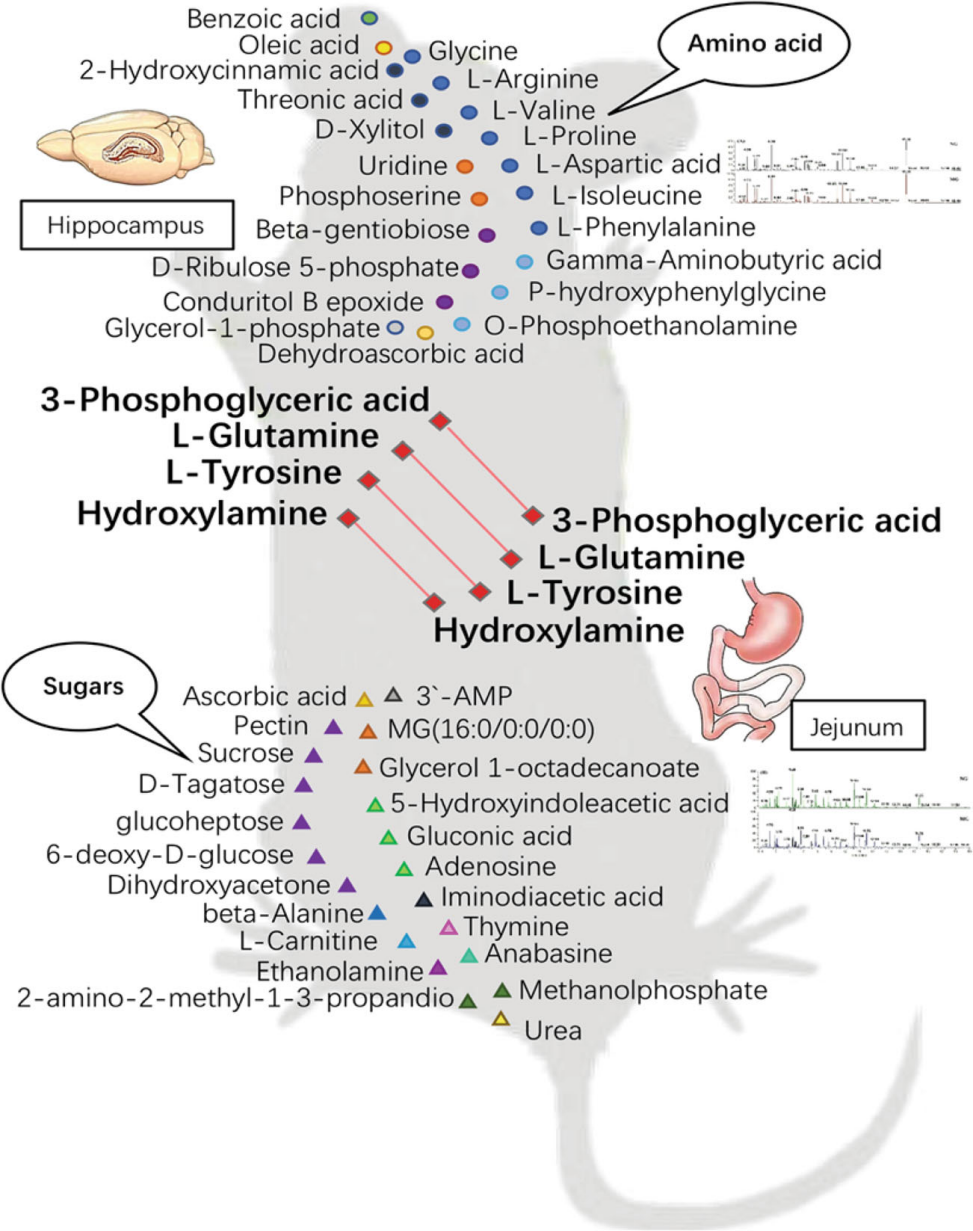
Schematic diagram of metabolite distribution. Metabolites derived from the amino acid difference in the hippocampus. Metabolites derived from the different metabolites of sugar in the jejunum. Different metabolites coexisting in the hippocampus and jejunum

**Fig. 8:**
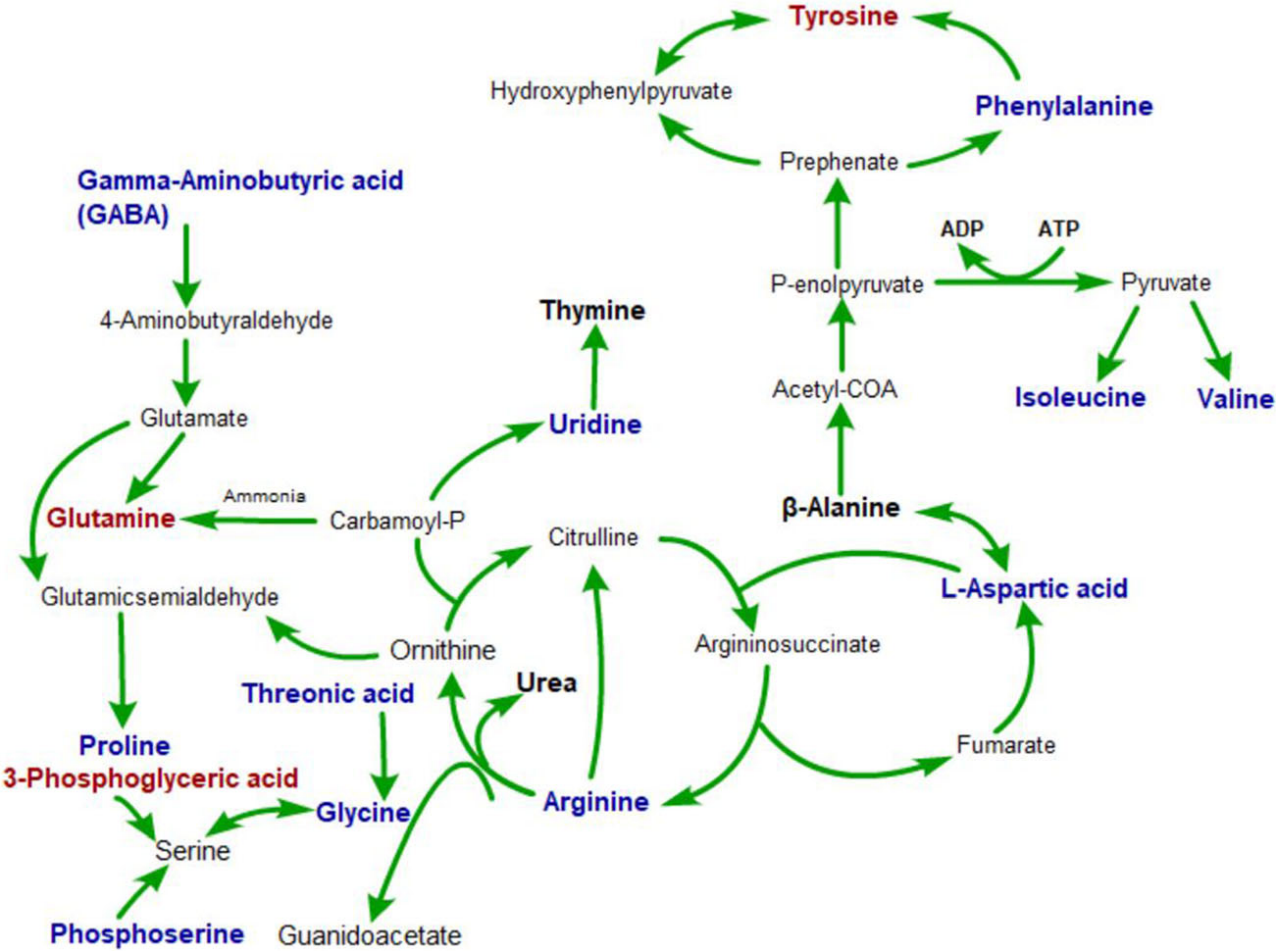
The pathways closely associated with the depression effects. Bold blue metabolites were potential biomarkers of the hippocampus from depression model; bold black metabolites were potential biomarkers of the jejunum from depression model. Bold red metabolites were potential biomarkers of the hippocampus and jejunum from depression model

These metabolite changes may be related to microbiota, which has emerged as a key player in control of the brain-gut axis, especially during stressful conditions provoked by real or perceived homeostatic challenge (Hashimoto et al. 2016; Jianguo et al. 2019). Many studies about gut-brain-gut signaling pathways pointed to metabolic changes, such as short chain fatty acids. Metabolites can serve as key messengers for the two-way communication system between the gut and the brain (Benech et al. 2021; Qiao et al. 2014). However, what molecular metabolite plays an important role in the process remains unknown (Kim et al. 2019a). The connection between the metabolic state of the intestines and the central nervous system may be a key to uncover the pathogenesis of depression. As far as we know, this is the first study to investigate the common metabolites affected by brain-gut interaction in depression-like behavior in rats. Various amino acids have been tested in the hippocampus and jejunum of UCMS rats, including proline, arginine, valine β-alanine, isoleucine, and gamma-aminobutyric acid. Out of the hundreds of metabolites detected in the hippocampus and jejunum samples, 26 metabolites levels were substantially changed and recommended as potential biomarkers for depression (Table 1 and Fig. 7). Using MetaboAnalyst 4.0, Kyoto Encyclopedia of Genes and Genomes (KEGG) (http://www.kegg.jp/), and Human Metabolome Database (HMDB) (http://www.hmdb.ca/), we conducted metabolic pathway analysis based on the distribution of differential metabolites, and a network of pathways of brain-gut axis metabolism in rats with UCMS-induced depression-like behavior was built with Pathway Builder Tool 2.0 (Fig. 8).

Monoaminergic neurotransmitters have proven to play a key role in the emotion-related disorders, and the first-line of treatments for these diseases are still targeting these neurotransmitters (Gu et al. 2016, 2018a, 2019, Liang et al., 2021, Xu et al., 2020). In our previous paper, we hypothesized that three monoamines might work as three substrates for the three primary basic emotions: DA-reward-joy, NE- stress (fear and anger), and serotonin-punishment (sad) (Wang et al. 2020, Gu et al. 2019). For example, DA is a catecholamine, which plays an important role in the brain reward system (Wang et al. 2018). Anhedonia, a characteristic feature of major depressive disorder, is often caused by the dysfunction of the reward system in the brain (Burlina et al. 2000). Tyrosine derived from liver phenylalanine (Phe) is a precursor of catecholamines (Ney et al. 2017; Huang et al. 2018), including NE, adrenaline (Ad), and DA. Lack of tyrosine not only leads to insufficient DA synthesis but also weakens the connection between striatum and frontal lobe region, which can induce cognitive impairment (Aleksidze and Blomstrand 1968; Lonart and Johnson 1995). These findings are consistent with the results of our study. Supplementation of tyrosine to individuals with phenylketonuria (PKU) increases the metabolic concentrations of DA and serotonin in cerebrospinal fluid with supplemental probiotics (Gross 1985; Kalmar et al. 2014), suggesting that tyrosine uptake may affect the synthesis of neurotransmitters. Depressive patients often have intestinal problems such as intestinal inflammation (Diamond 1983), and intestinal damage will inevitably impair tyrosine intake. This again proves the correlation between depression and intestinal damage.

Nitric oxide (NO) derived from hydroxylamine and S-nitroso-L-cysteine (NO-CYS) can evoke release of hippocampal NE (Morikawa et al. 2007). Being a product of cell metabolism, hydroxylamine, however, can be toxic when it reaches certain level. In brain tissue, it strongly stimulates the oxidation of succinic acid in neurons (Kim et al. 2019b), activates guanylate cyclase in the granule part of cerebral cortex, and inhibits the enzyme activity in glial cells. Hydroxylamine and its derivatives can be neuroprotective and prevent convulsions by suppressing the GABA-transaminase in many neurodegenerative disease models (Glade and Smith 2015; Sabogal-Guáqueta et al. 2018). Normal concentrations of hydroxylamine are associated with the maintenance of normal neural responses in the brain. The hydroxylamine class has also been shown to relax nonvascular smooth muscle at an effective concentration (Edogawa et al. 2020), which can explain the frequent co-occurrence of severe depression (MDD) and irritable bowel syndrome (IBS), as MDD is associated with decreased level of hydroxylamine in intestines.

3-Phosphoglyceric acid is a precursor for serine, which is an endogenous ligand of NMDA-R. Serine concentration in mouse cerebellum is very low (Fernandes and Gupta 2019); however, no change in serine was found in the hippocampus of UCMS rats in this study. The feasibility of using the precursor 3-phosphoglyceric acid content in the hippocampus instead of its content for testing is unknown, but it is certain that L-serine can protect neuronal cells against oxidative stress–mediated apoptotic cell death by contributing to intracellular antioxidant GSH synthesis and maintaining the mitochondrial fusion-fission balance (Qian et al. 2019). It can support human cognitive functions, including formation of short-term memory, consolidation of long-term memory, learning and recalling information, and development of language and communication skills. Human brain uses serine to synthesize phosphatidylserine. Phosphatidylserine is closely related to aging of human brain, biochemical changes in neurotransmission, and structural deterioration (Cajka et al. 2017). In the case of cerebral ischemia, phosphatidylserine deficiency in the hippocampus leads to cognitive impairment (Tsugawa et al. 2015). All in all, this is related to the content of 3-phosphoglyceric acid in rats with depression-like behavior in this study, suggesting that there is some damage to neurons in the brain. Increases of proteolytic activity (PA) in the intestine can disrupt the intestinal shield and increase the intestinal stress response. Serine protects the intestine by inhibiting protease-activated receptor 2 (Par-2) (Xi et al. 2014). The increase in the content of 3-phosphoglycerate related to serine synthesis in this study may be a negative feedback regulation of disease in the intestine of the body. It is speculated that in the UCMS model, substances in the hippocampus have corresponding changes in jejunal metabolism.

Emotional and cognitive impairment and altered hippocampal synaptic plasticity are important features of depression (Tsugawa et al. 2015). Synaptic plasticity is known to regulate and support signal transduction between neurons, and synaptic dysfunction leads to a variety of neurological disorders. Exposure to chronic unpredictable mild stress stimuli in rats for a period of time has led to obstruction of information transmission between glial cells and neurons and decrease of LTP induction at the CA3 to CA1 synapses of the hippocampus (Xi et al. 2014; L’Huillier et al. 2019). These changes in structure and plasticity will further impair hippocampus-dependent learning and memory functions (L’Huillier et al. 2020). Glutamine is a precursor for glutamate, which is the main excitatory neurotransmitter in the brain, and serves as the neurotransmitter between endocrine cells and neurons (Kong et al. 2018). The LTP of CA3-CA1 in the hippocampal synapse requires glutamate-mediated activation of NMDA receptor production (Gracie et al. 2019). Intervention of the glutamate system may be the underlying antidepressant mechanism of ketamine, which is a non-competitive antagonist of the N-methyl-D-aspartate (NMDA) type of glutamate receptor (Zamani et al. 2019). In this study, we found that metabolic disorder of glutamine-glutamic acid cycle in brain-gut network is related to the LTP changes. Traditionally treated as a nutritious nonessential amino acid, glutamine is now recognized as a key player in activating the mammalian target of rapamycin cell signal transduction in intestinal cells, promoting proliferation and survival of intestinal cells, repairing intestinal mucosa and maintaining intestinal integrity under stress, and protecting the intestinal tract from atrophy and injury (Gracie et al. 2019; Mahar et al. 2021). Changes in glutamine metabolism in the intestine can induce intestinal shielding dysfunction (LaVoie et al. 2005) and regulate the homeostasis (differentiation, proliferation, renewal) and immune response of intestinal epithelium. The decrease of glutamine content in the intestine and brain could explain that psychological disorder is associated with recurrence of intestinal disease (Spies et al. 2020) and that the incidence of depression in patients with intestinal disease is three times higher than that in normal people (Hensel et al. 2019). Additionally, growing evidence suggests that glutamine holds great potential for being a necessary amino acid supplement in the treatment of depression.

## Conclusion

In conclusion, our study suggests that UCMS-induced depression-like behavior in rats displayed inhibited LTP of Schaffer collateral-CA1 synapses in the hippocampus and metabolic alterations of the brain-gut axis. The underlying mechanisms for this association and how these metabolic alterations interact with synaptic plasticity warrant further investigation. This study is expected to shed light on the neurobiology of depression and will guide the development of new treatment for this menacing neuropsychiatric disorder.

## Supplementary information

ESM 1 (PDF 178 kb)

ESM 2 (PDF 783 kb)
