# Supplementary material for: Metabolomics changes in brain-gut axis after unpredictable chronic mild stress
Source: Psychopharmacology (Berl). 2022 Feb 8;239(3):729–43. doi: 10.1007/s00213-021-05958-w (PMC8891102; doi:10.1007/s00213-021-05958-w)
Supplement: Supplementary file 1 — (PDF 178 kb) [file 213_2021_5958_MOESM1_ESM.pdf]

Supplementary figures. "Fushun Wang. *, Qiuyue Xu, Mingchen Jiang, Simeng Gu, Xunle Zhang, Fushun Wang, Erxi Wu, Jason H Huang" **Metabolomics Changes in Brain-gut Axis in Chronic Unpredictable Mild Stress Induced Depressive Rats**

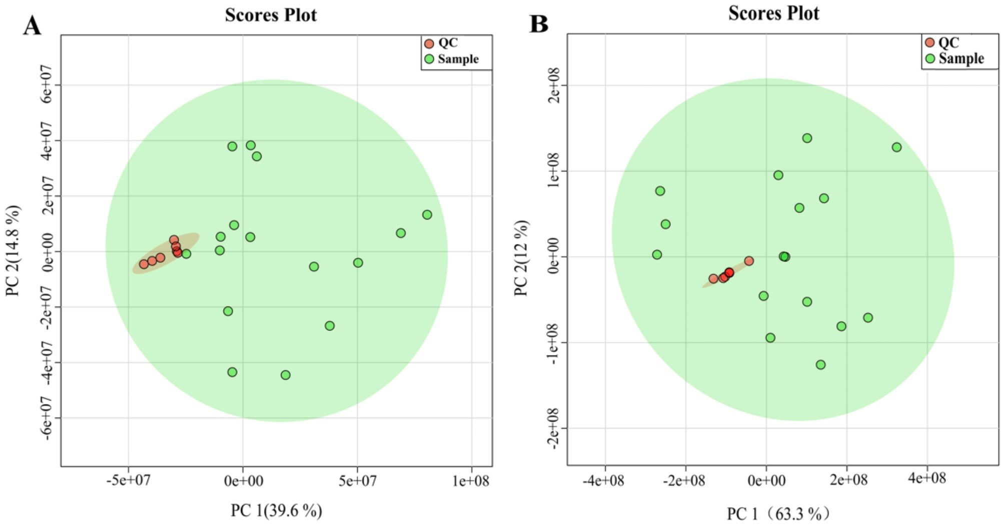

Figure S1. PCA score plots of metabolic profiling of QC and experimental samples in the hippocampus and jejunum samples. (A) QC aggregates in the same position shows that the stability of the instrument is better when detecting hippocampal samples. (B) QC aggregates in the same position shows that the stability of the instrument is better when detecting jejunum samples.
