# Supplementary material for: Metabolomics changes in brain-gut axis after unpredictable chronic mild stress
Source: Psychopharmacology (Berl). 2022 Feb 8;239(3):729–43. doi: 10.1007/s00213-021-05958-w (PMC8891102; doi:10.1007/s00213-021-05958-w)
Supplement: Supplementary file 2 — (PDF 783 kb) [file 213_2021_5958_MOESM2_ESM.pdf]

Supplementary figures. “Fushun Wang. *, Qiuyue Xu, Mingchen Jiang, Simeng Gu, Xunle Zhang, Fushun Wang, Erxi Wu, Jason H Huang” **Metabolomics Changes in Brain-gut Axis in Chronic Unpredictable Mild Stress Induced Depressive Rats**
” **Metabolomics Changes in Brain-gut Axis in Chronic Unpredictable Mild Stress Induced Depressive Rats**

In order to verify the correlation between the jejunum and hippocampus and the metabolite content in serum, the metabolite profile of serum samples was continued to be studied by GC-MS. In serum samples, 101 endogenous metabolites were identified. After the data was normalized, QC was used to test the stability of the samples (**Figure S2 A**), and the correlation between the groups was determined by principal component analysis. The normal group and the model group can be separated (**Figure S2 B**). The differential metabolites were selected based on one-way ANOVA and q test ($p < 0.05$), and there was a fold change> 1.2 between the normal group and CUMS model group (**Figure S3**). For research purposes, we focused on the common metabolites the in the hippocampus and the jejunum, L-Glutamic acid and L-Tyrosine have metabolic changes in peripheral serum that are slightly different from those in the hippocampus and jejunum (**Figure S4**).
Tyrosine is an essential amino acid that easily crosses the blood-brain barrier and may be detrimental to mental illness[1]. Many antipsychotic drugs apparently work by inhibiting tyrosine metabolism[2]. Glutamate has a role in mediating the production of the nervous system Rapid inhibitory effects[3]. L-Glutamic acid is a glutamine product Its content in the serum of the CUMS model group is reduced, positive correlation with previous the hippocampus and jejunum experimental results. The L-Tyrosine in the serum is opposite to that in the hippocampus and jejunum, which indicates that the consumption of L-Glutamic acid by the disease may be systemic, while L-Tyrosine It may be caused by local cell depletion or absorption disorders, these still require further verification.

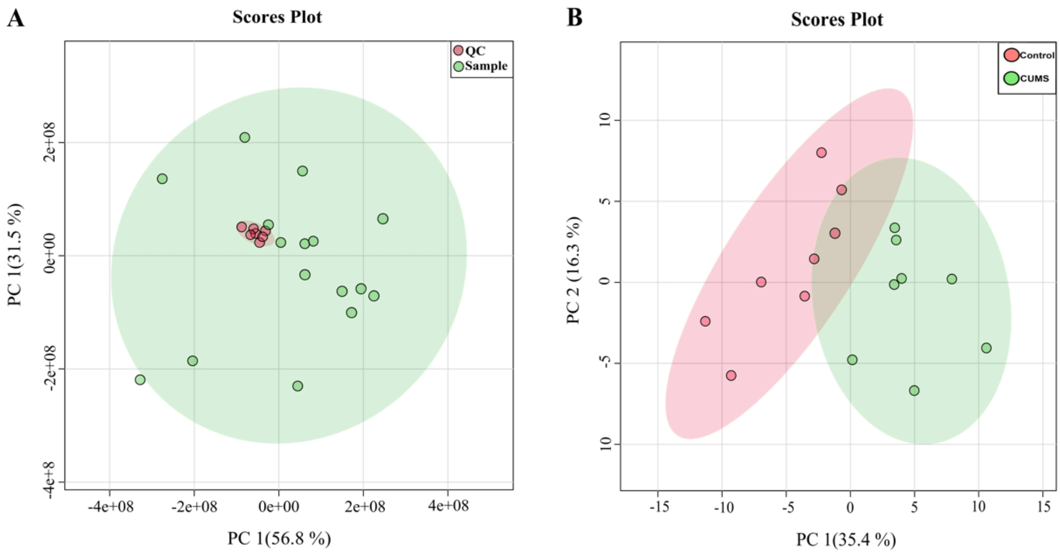

Figure S2. PCA score plots of metabolic profiling of QC and experimental samples in (a) serum samples. PCA scores scatter plot of (b) serum metabolites in control and CUMS.

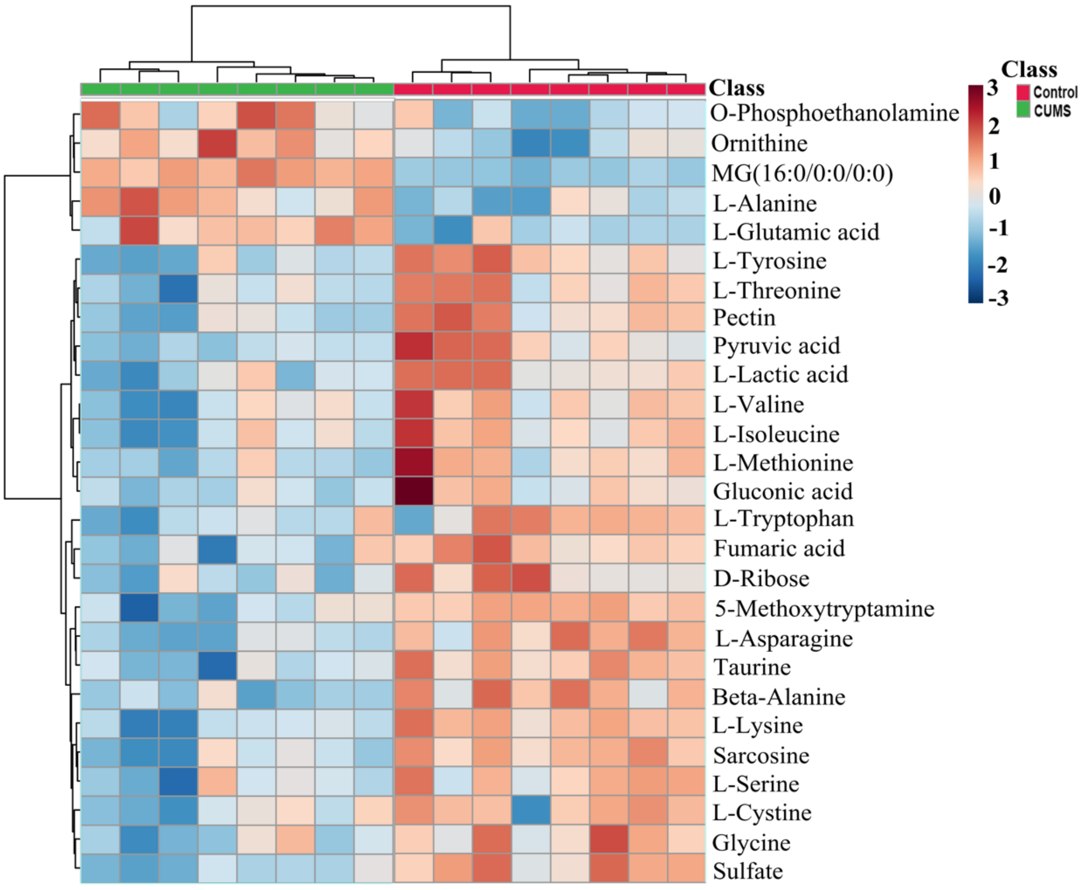

Figure S3. Heatmap of identified differential metabolites with serum metabolomics profile.

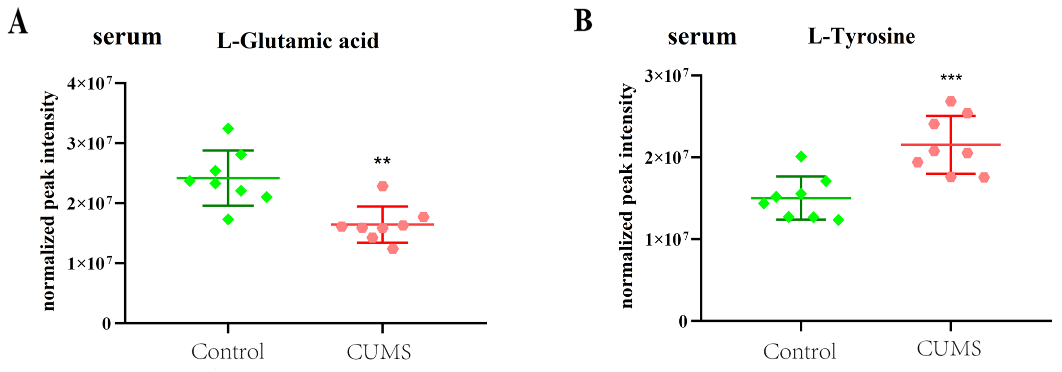

Figure S4. Scatter plots of significantly changed metabolites normalized peak intensity in rat serum samples. The x-axis shows the specific metabolite's normalized peak intensity, and each scatter represents a corresponding sample of the rat. **p＜0.01, ***p＜0.001, *vs.* control.

References:

1. Sogawa Rintaro,Kimura Sakiko,Yakabe Ryota et al. Anxiety and depression associated with tyrosine kinase inhibitor discontinuation in patients with chronic myeloid leukemia.[J] .Int. J. Clin. Oncol., 2018,

23: 974-979.
2. Waldmeier P C,Effects of chronic antidepressant treatment on pre- and post-synaptic dopaminergic mechanisms. A short review of the literature and some complementary experiments. [J]. Pol J Pharmacol Pharm, 1984, 36: 201-16.
3. Lener Marc S,Niciu Mark J,Ballard Elizabeth D et al. Glutamate and Gamma-Aminobutyric Acid Systems in the Pathophysiology of Major Depression and Antidepressant Response to Ketamine.[J] .Biol. Psychiatry, 2017, 81: 886-897.
